# Predictors for Gingival Index in Middle-Aged Asian Indians with Type 2 Diabetes from South India: A Cross-Sectional Observational Study

**DOI:** 10.1155/2018/9081572

**Published:** 2018-01-02

**Authors:** S. Jai Karthik, Shajith Anoop, R. Suresh Kumar, M. V. Usha Rani

**Affiliations:** ^1^Department of Environmental Sciences, Bharathiar University, Coimbatore, Tamil Nadu, India; ^2^RVS Dental College, Coimbatore, Tamil Nadu, India; ^3^Suresh Diabetes Centre, Vadavalli, Coimbatore, Tamil Nadu, India

## Abstract

Asian Indians develop type 2 diabetes mellitus (T2DM) much earlier as compared to White Caucasians, due to unique phenotypic and genetic architecture. Periodontitis in T2DM patients is often a neglected clinical feature. This study was conducted to derive predictor variables for gingival index in middle-aged Asian Indians with T2DM in a semiurban population of Dravidian ethnicity from Tamil Nadu, India. T2DM patients (*n* = 232, mean age: 50.6 ± 10.4 years) with periodontitis (*n* = 123, mean age: 54.3 ± 2.4 years) and without periodontitis (*n* = 109, mean age: 55.2 ± 3.1 years) were recruited between 2014 and 2016 by purposive sampling method. Dental examinations for pocket depth (PD) and clinical attachment level (CAL) were performed and gingival index was calculated. Fasting venous blood samples were analysed for measures of glycaemia and cholesterol. Significant positive correlation (*p* < 0.01) was observed for gingival index with glycosylated haemoglobin (HbA1c), pocket depth, presence of T2DM, and clinical attachment level. Stepwise multiple linear regression analysis derived increased pocket depth (*p* < 0.01), elevated HbA1c (*p* < 0.01), clinical attachment level (*p* < 0.01), and presence of diabetes (*p* < 0.01) as significant predictors (*r* ^2^ value = 0.67) for increased gingival index in middle aged patients with T2DM. These variables significantly (*p* < 0.01) predispose middle-aged T2DM patients to increased gingival index, thus warranting appropriate intervention.

## 1. Introduction

The incidence of type 2 diabetes mellitus (T2DM) is ever growing in Southern Asia and is predicted to increase along with urbanization [1]. Among other secondary complications [2, 3] a close link exists between T2DM, periodontitis, and liver diseases [4]. Importantly, periodontal disease is associated with cardiovascular diseases and is predictive of future cardiac events [5]. A bidirectional relationship exists between T2DM and periodontal disease [6] which is attributed to persisting hyperglycemia, leading to an exaggerated immuno-inflammatory response to pathogenic microbial challenge in the gingiva [7]. In T2DM, persisting hyperglycemia causes non-enzymatic glycation and oxidation of proteins and lipids, leading to the subsequent formation of advanced glycation end products (AGEs) [8]. AGEs with accompanying markers for increased oxidative stress have been demonstrated in human gingiva in T2DM subjects with periodontitis, leading to rapid and severe periodontal tissue destruction [9].

Importantly, dental health in T2DM patients is indicative of glycaemic control and can also predict future risk of retinopathy and neuropathy [10]. With the increasing prevalence of T2DM in Asian Indians and its comorbidities including periodontitis, it is alarming to note that dental health in T2DM patients, especially the middle aged, is much neglected.

We hypothesized that glycaemic status in T2DM patients is closely related to gingival index and aimed to determine the clinical variables that correlate with gingival index in a sample of middle aged T2DM subjects with and without chronic periodontitis from a semiurban population of South India. We aimed to derive simple predictors that can be applied for early detection of periodontal risk in T2DM patients aged above 50 years, such that preventive therapy can be initiated.

## 2. Methods

This cross-sectional study was conducted in a dental care centre at Coimbatore, Tamil Nadu. Ethical approval was obtained from the Institutional Ethics Committee of RVS Dental College, Coimbatore, prior to the commencement of the study. The objectives were explained to all participating subjects and signed and written consent was obtained prior to enrollment in the study.

### 2.1. Subject Population and Selection

Purposive convenient sampling method was adopted. A minimum sample size of 80 subjects for each arm was determined with an alpha error of 5% at 90% power (95% CI). A total of 232 middle-aged (above 50 years of age) patients with T2DM were recruited. Inclusion criteria were presence of clinically diagnosed T2DM at least 12 months prior to the start of the study and at least 4 fully erupted teeth. A questionnaire based interview was administered to participants to assess awareness of dental health and hygiene. Quality of dental hygiene was determined as frequency and method of brushing, frequency of tooth brush change, dental flossing, number of dental visits for scaling per year, habitual chewing of tobacco, and pyorrhea. Clinical history of patients was obtained with consent from the treating physician and the patient. T2DM subjects with chronic history of smoking, alcoholism, dental abscesses, overt pyorrhea, use of dental braces, and capping or missing teeth were excluded from the study.

### 2.2. Study Protocol

Periodontal examinations were performed using sterile instruments by a single examiner for all study subjects. Pocket depth (PD) measurements were obtained by using William's periodontal probe. The PD was measured from the free gingival margin to the base of the pocket. The probe was maintained parallel to the long axis of the tooth. Pocket depth and clinical attachment level (CAL) were measured for each tooth at 6 sites, namely, mesiobuccal, midbuccal, distobuccal, mesiolingual, midlingual, and distolingual. Clinical attachment level (CAL) was determined by measuring the distance from the cement enamel junction (CEJ) to the gingival margin with a William's periodontal probe. Gingival index (GI) was determined and recorded at 4 gingival sites per tooth using the following criteria: (0) normal gingiva, (1) mild gingivitis without bleeding on probing, (2) moderate gingivitis with bleeding on probing, and (3) severe gingivitis with ulceration and spontaneous bleeding. The sum of the scores from the four areas of each tooth was divided by 4 to derive the gingival index for that tooth. The mean GI score was obtained after calculating individual GI [11].

Fasting blood glucose (FBG) after an eight-hour overnight fast, 120 min postprandial blood glucose (PPBG), glycosylated hemoglobin (HbA1c), and serum cholesterol were analysed for all subjects on the same day. FBG and PPBG were analysed by glucose-oxidase peroxidase method. HbA1c was estimated by high performance liquid chromatography and expressed as percentage value. Serum lipid profile was measured by methods mentioned in a previous study from our group [12]. Overweight and obesity were defined in accordance with the cut-offs for Asian Indians [13]. Dyslipidemia was defined by the criteria formulated by the National Cholesterol Education Program, Adult Treatment Panel III (NCEP–ATP III) [14]. T2DM was diagnosed if fasting plasma glucose was ≥126 mg/dL and/or 120 min postprandial plasma glucose was ≥200 mg/dL or there was self-reported medication for diabetes by the participant [15].

## 3. Data Analysis

Statistical analysis was performed using statistical software STATA (version 14.2, StataCorp, College Station, Texas, USA). Data was checked for normative distribution and an independent samples* t*-test was applied for continuous variables. Pearson's correlation test and stepwise multiple linear regression (SMLR) analysis were used to analyse correlations and to derive predictor variables, respectively. The *p* value ≤ 0.05 was considered as statistically significant.

## 4. Results

The sample size was matched for age and BMI. Of the 232 obese, middle aged T2DM subjects recruited in this study, 123 subjects with T2DM (53.01%) had no indications of periodontitis whereas 109 subjects (46.55%) with T2DM had chronic periodontitis. The mean values of BMI, FBG, HbA1c, mean pocket depth, and gingival index were comparatively higher in T2DM patients with significant differences in clinical attachment level (CAL), probing depth (PPD), and gingival index (Table 1). Genderwise comparisons revealed no significant differences in the clinical profile, clinical attachment level, gingival index, and pocket depth (Table 2).

**Figure 1:**
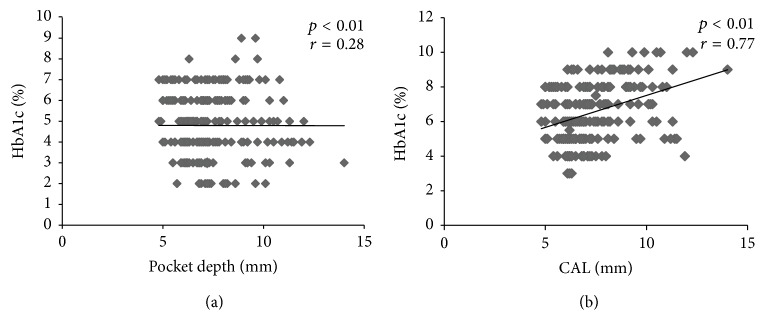
(a) Scatter plot showing correlation between HbA1c and pocket depth. (b) Scatter plot showing correlation between HbA1c and clinical attachment level (CAL).

In this study, patients with T2DM and periodontitis had periodontal pockets measuring at least 4 mm deep with bleeding on probing and radiographic evidence of bone loss above 50%. T2DM patients without periodontitis had periodontal pockets measuring less than 4 mm deep with no radiographic evidence of bone loss. Correlation statistics revealed significant positive correlation (*p* < 0.001) between gingival index, pocket depth, presence of diabetes, and clinical attachment level (Table 3). Significant positive correlation was also observed between HbA1c and pocket depth (Figure 1(a)) and clinical attachment level (Figure 1(b)). Irrespective of age and gender, the predictive variables for gingival index in the study cohort were HbA1c (*p* < 0.01), pocket depth (*p* < 0.01), and presence of diabetes (*p* < 0.01), with a regression coefficient (*r* ^2^) value of 0.67 (Table 4).

Genderwise correlations revealed significant positive correlation of gingival index with age, (*p* < 0.001), BMI (*p* < 0.001), HbA1c (*p* < 0.001), pocket depth (*p* < 0.001), and clinical attachment level (*p* < 0.001) in males. However, though significant correlations were observed for HbA1c, pocket depth (*p* < 0.001), and clinical attachment level (*p* < 0.001), no significant correlation was observed for gingival index with age and BMI in females (Table 5).

Gender based analysis for predictors of gingival index revealed pocket depth and clinical attachment level as significant determinants in males and females (Table 6).

We analysed the data for correlations on the basis of medications, namely, OADs (metformin + sulphonylureas) (Group 1) and insulin (Group 2). For T2DM patients on OADS, significant positive correlation was observed for gingival index with fasting blood glucose (*p* < 0.05), triglycerides (*p* < 0.01), pocket depth (*p* < 0.001), and clinical attachment level, but significant negative correlation was observed with age (*p* < 0.01). Interestingly, in T2DM patients on insulin therapy, significant positive correlation was observed for gingival index with HbA1c (*r* = 0.21, *p* < 0.01), pocket depth (*r* = 0.57, *p* < 0.001), and clinical attachment level (*r* = 0.73, *p* < 0.001) but not for fasting blood glucose and triglycerides (Table 7). Above all, SMLR analysis for predictors of gingival index based on medications did not derive gingival index, pocket depth, and clinical attachment as significant determinants.

## 5. Discussion

Systemic inflammation is significantly elevated in the presence of obesity, insulin resistance, hyperglycemia, and T2DM which derange the normal metabolic and endocrine functions of adipose tissue, resulting in increased production of fatty acids, hormones, cytokines, and acute phase reactants [16]. T2DM is a risk factor for gingivitis and periodontitis while the level of glycaemic control appears to be an important determinant in this relationship [17]. In our study, significant differences in gingival index were observed between the two groups. The increased levels of attachment loss and pocket depth in T2DM subjects with periodontitis are similar to observations from recent studies [18, 19]. Further, we observed that gingival index is positively associated with glycosylated haemoglobin despite lack of significant differences in blood glucose levels in both groups. In another case-control study, positive associations between HbA1c and CAL and BOP were observed suggesting the need for rigorous glycaemic control to alleviate periodontitis in patients with T2DM [20].

In the present study, three predictive factors for gingival index, namely, HbA1c, pocket depth, and presence of diabetes, were obtained. A combination of these factors leads to elevated risk of periodontitis upto 67% in middle aged subjects with T2DM. Genderwise comparisons revealed significant correlation of gingival index with HbA1c, pocket depth, and CAL in males and females. Specifically, a higher degree of correlation was observed in females for pocket depth and clinical attachment. Interestingly, pocket depth, clinical attachment level, and HbA1c served as strong predictors of gingival index, irrespective of gender. Similarly, a study on Korean T2DM patients (aged 40−70) showed HbA1c and fasting plasma glucose as strong predictors of periodontal health, irrespective of gender [21]. The presence of obesity and hyperglycaemia aggravates gingival inflammation while optimal adiposity and glycaemic control leads to a concomitant reduction in gingival inflammation [22]. Well defined studies have demonstrated that T2DM subjects with periodontitis are highly prone to diabetic nephropathy [23], diabetic retinopathy, and ketoacidosis than T2DM subjects without periodontitis [24]. In this context, our observations gain importance as gingival index and HbA1c can be used together for better monitoring of elderly T2DM patients for secondary complications of diabetes.

Considering clinical management for T2DM, we observed no significant differences between T2DM patients treated by oral metformin/sulphonylureas and T2DM patients on insulin. This may be due to the fact that this is an observational study and a prospective follow-up study is required to delineate the effects of medications on periodontal health. In addition, patients in our study were age and BMI matched with no overt microvascular and macrovascular complications during the course of the study. This could have resulted from an absence of differential effects, due to antihyperglycemic therapy. It would be interesting to observe the differential effects of locally delivered subgingival metformin therapy and oral metformin in Asian Indians with T2DM, as done in an earlier study elsewhere [25].

It is important to note that in patients with T2DM, social factors can significantly impact oral health status and access to dental care. A large epidemiological survey reported that people with lower incomes had poorer oral health status especially as the type of oral healthcare provided was rarely preventive [26]. Importantly in this study we observed that T2DM patients of both groups did not practise brushing before bed time and habitual dental flossing. Most of the subjects reported having a dental examination only during tooth ache or intensive gum bleeding. We also observed that the study subjects lacked awareness of the importance of dental health probably due to less emphasis on dental health in clinical care of T2DM. This is similar to the observations of another study which reported T2DM as an important risk factor for periodontitis [27]. Furthermore, most T2DM patients reported that their treating physicians did not emphasize the need for a dental checkup as in semiurban India and dental care is available only as a separate speciality and is not part of a comprehensive diabetes care regimen. In view of such scenarios, the predictors derived in our study hold robust predictive value (*r* ^2^ value = 0.67) and can be effectively applied for screening and prevention of periodontal complications. Further, considering the social scenario of the study population, it would be very ideal if physicians treating patients with T2DM also advise them on periodic dental examinations and provide accessible dental care at affordable costs. We acknowledge the limitations of this study as being cross-sectional and non-interventional in design. Also, the correlation of periodontitis with secondary complications of T2DM was not studied. In this context, prospective, large scale studies are required in different parts of India to validate the observations. Nevertheless, this study has reported three simple and important variables that predict the risk of severe periodontitis in middle aged, obese subjects with T2DM which can be applied in other populations of Tamil Nadu.

With ever increasing burden of T2DM in India, dental care and screening programs need to be scaled up with sustained treatment regimens at affordable costs. A comprehensive dental care program for populations of different socioeconomic strata needs to be envisaged. Interventional studies at low cost need to be conducted to ameliorate and manage dental complications in T2DM.

## 6. Conclusion

Glycosylated hemoglobin, pocket depth, and clinical attachment level can be used in comprehensive screening of T2DM patients for periodontitis and for initiation of preventive therapy.

## Figures and Tables

**Table 1:** Descriptive statistics between groups.

Demographic & clinical profile	Group 1 (*n* = 123)	Group 2 (*n* = 109)	*p* value
Age (years)	54.3 ± 2.4	55.2 ± 3.1	0.28
Body mass index (kg/m^2^)	25.9 ± 2.5	26.4 ± 3.5	0.23
Fasting blood glucose (mg/dl)	126.07 ± 45.4	131.2 ± 63.7	0.47
Postprandial blood glucose (mg/dl)	187.8 ± 78.2	182.34 ± 78.5	0.58
Glycosylated haemoglobin (%)	7.1 ± 1.3	7.7 ± 1.8	<0.05
Total cholesterol (mg/dl)	229.7 ± 43.1	218.28 ± 42.3	<0.05
High-density lipoprotein cholesterol (mg/dl)	40.29 ± 8.9	42.45 ± 8.7	0.06
Low-density lipoprotein cholesterol (mg/dl)	146.74 ± 34.4	137.97 ± 33.4	0.05
Triglycerides (mg/dl)	147.15 ± 50.6	147.66 ± 50.0	0.94
Clinical attachment level (mm)	5.38 ± 1.2	7.78 ± 1.1	<0.01
Pocket depth on probing (mm)	3.91 ± 1.2	5.79 ± 1.4	<0.01
Gingival index	1.83 ± 0.4	2.86 ± 0.3	<0.01

*p* < 0.05: statistically significant; *Group 1*: T2DM subjects without periodontitis; *Group 2:* T2DM subjects with periodontitis.

**Table 2:** Descriptive statistics across gender.

Demographic & clinical profile	Males (*n* = 130)	Females (*n* = 102)	*p* value
Age (years)	50.1 ± 10.2	51.1 ± 10.7	0.48
Body mass index (kg/m^2^)	26.1 ± 2.3	26.1 ± 3.7	0.90
Fasting blood glucose (mg/dl)	127.6 ± 50.0	129.5 ± 60.4	0.78
Postprandial blood glucose (mg/dl)	188.5 ± 77.1	181.1 ± 76.8	0.47
Glycosylated haemoglobin (%)	7.3 ± 1.7	7.4 ± 1.5	0.85
Total cholesterol (mg/dl)	224.9 ± 43.9	223 ± 42.6	0.81
High-density lipoprotein cholesterol (mg/dl)	41.9 ± 9.0	40.3 ± 8.6	0.19
Low-density lipoprotein cholesterol (mg/dl)	145.6 ± 35.2	138 ± 32.4	0.13
Triglycerides (mg/dl)	148.1 ± 53.4	146.4 ± 46.1	0.80
Clinical attachment level (mm)	6.6 ± 1.6	6.3 ± 1.7	0.28
Pocket depth on probing (mm)	4.8 ± 1.4	4.7 ± 1.6	0.90
Gingival index	2.3 ± 0.6	2.4 ± 0.6	0.69

*p* < 0.05: statistically significant.

**Table 3:** Correlation of clinical parameters with gingival index in the study cohort.

Clinical variable	Correlation coefficient	*p* value
Body mass index (kg/m^2^)	0.13	NS
Glycosylated haemoglobin (%)	0.28	<0.01
High density lipoprotein cholesterol (mg/dl)	0.16	NS
Presence of diabetes	0.78	<0.01
Clinical attachment level (mm)	0.77	<0.01
Pocket depth (mm)	0.63	<0.01

*p* < 0.05: statistically significant*; NS: not significant.*

**Table 4:** Predictors of gingival index in patients with T2DM.

Predictor variables	Standardized coefficient (beta value )	*p* value	95% confidence interval
Glycosylated haemoglobin (%)	0.16	<0.01	0.007–0.058
Pocket depth (mm)	0.24	<0.01	0.19–0.27
Presence of diabetes	0.59	<0.01	0.08–0.15

*p* < 0.05: statistically significant;regression coefficient value: (*r* ^2^) = 0.67.

**Table 5:** Gender specific correlations of clinical variables with gingival index.

Variables	Correlation coefficient		*p* value	
	Males	Females	Males	Females
Age (years)	0.30	0.05	<0.001	0.60
BMI (kg/m^2^)	0.27	0.02	<0.01	0.77
HbA1c (%)	0.30	0.26	<0.001	<0.01
Pocket depth (mm)	0.57	0.63	<0.001	<0.001
Clinical attachment level	0.77	0.76	<0.001	<0.001

*p* < 0.05: statistically significant.

**Table 6:** Gender specific predictors of gingival index in patients with T2DM.

Predictor variables for gingival index	Standardized coefficient (beta value)		*95% confidence interval*		*p* value	
	Males	Females	Males	Females	Males	Females
BMI (kg/m^2^)	0.06	0.00	0.03, 08	−0.02, 0.02	<0.001	0.87
HbA1c (%)	0.006	0.03	0.03, 0.04	−0.02, 0.92	0.75	0.29
Pocket depth (mm)	0.10	0.12	0.05, 0.15	0.05, 0.19	<0.001	<0.001
Clinical attachment level	0.24	0.21	0.19, 0.29	0.15, 0.28	<0.001	<0.001

*p* < 0.05: statistically significant*; *regression coefficient value: (*r* ^2^) = 0.63.

**Table 7:** Correlation of gingival index with clinical variables based on antihyperglycemic medications.

Variables	OADs		Insulin	
	Correlation coefficient	*p* value	Correlation coefficient	*p* value
Fasting blood glucose	0.41	<0.05	0.13	0.05
BMI (kg/m^2^)	0.05	0.76	0.03	0.6
HbA1c (%)	0.05	0.78	0.21	<0.05
Triglycerides (mg/dl)	0.47	<0.01	0.06	0.35
Pocket depth (mm)	0.73	<0.001	0.57	<0.001
Clinical attachment level	0.51	<0.01	0.73	<0.001

*p* < 0.05: statistically significant.
